# Reflections on Binary Sex/Gender Categorization in Magnetic Resonance Tomography and its Future Challenges

**DOI:** 10.3389/fsoc.2021.705106

**Published:** 2021-07-21

**Authors:** Hannah Fitsch

**Affiliations:** Technische Universität Berlin, Berlin, Germany

**Keywords:** brain imaging, fMRI, sex/gender binary, interdiscipinary research, taxonomy, intersectionality

## Abstract

This paper examines the role of technical, methodological conditions in functional magnetic imaging (fMRI) in the production of binary sex/gender differences. The aim is to investigate the scanning process with a focus on the statistical parameter of gendered markers within the technology, in order to make visible the problems entangled in typical research routines. It is especially important to elaborate this because the computer models currently being used and Big Data studies are reproducing and reapplying outdated and rigid concepts of sex/gender differences with the goal of improving science considerably. Therefore, the paper discusses the empirical methodologies and epistemic underpinnings of differentiation through statistics, and argues that counter-counting, weighing and sizing might not help to substantiate the idea of “equality” (not only for the sex/gender category) in brain studies. In relation to the topic of this special issue, I argue that in order to develop an interdisciplinary approach to criticizing dimorphism and differentiation by groups, a wider understanding of the technical and theoretical foundations used in brain research is needed.

## Introduction

Before conducting my fieldwork in brain imaging labs, I assumed that the practice of gender categorization was set when the tomograph is programmed and that it directly affects the scanning process. I thought this mainly because, in practice, the first action of every scanning process is to set a marker: to indicate whether the person in the scanner is male or female. The third option, “neutral” is virtually never set when measuring human beings. It turned out that this specific moment in the scanning process is not when male/female markers become efficacious, or at least this moment does not necessarily inscribe the binary markers into the data. But of course, there is no such thing as “raw data”. “Raw Data” is an oxymoron, as Lisa Gitelman (2013) reminds us: “Every discipline and disciplinary institution has its own norms and standards for the imagination of data, just as every field has its accepted methodologies and its evolved structures of practice”. (Gitelman 2013, 3) Data is never just there, it needs to be generated, meaning that every object of investigation needs to be placed under a research issue and the assumptions of the method that is being used. In fMRI one of the most crucial underlying conditions is the concept of mapping, which falls into the tradition of categorization and specific thresholds plus values of normalization for groups. In fMRI most of the pre-processing and normalization steps are part of an “automated evaluation” built on digital atlases, which Anne Beaulieu has called “database diagnosis” (Beaulieu 2001, 664).

The preference for mapping and measuring differences in the brain has a long history. It has existed since the early days of brain research, when skull shapes were measured and rated by size and intelligence, as white, middle-class men were considered more intelligent than women and were also believed to be equipped with greater intelligence than was found in all other human groups and classes. This measurement of brains, or rather the mismeasurement of man (Gould 1996) led, given the doctrine of the normalization society, to standardizations and stereotypes. However, at all times there was also a critique of biological sex dimorphism, hierarchization and essentialism. Take, for example, the feminist (or suffragette, the term used in the 1880s) Helen H. (Gardener, 1887). (1853–1925) and her conviction that young girls “brains were conditioned in the same way as boys”, and therefore girls should have the same access to education. In her paper “Sex and brain weight” (1887) Gardener argued that no connection between brain weight and intellectual capacity had been proven, and she thus challenged the prevailing methodology for measuring brain size. Gardener’s approach to asserting the equality of male and female brains was based on the assumption that it was not the comparison that was problematic, but rather the basis of the comparison, and in her view this meant that brains from the same “race”^1^ and the same class perform equally. This idea of an “evolutionary ladder” was also part of Gardener’s approach. In her understanding not all women were equal, but some woman were more equal to well-educated white men than others. “The idea that brains could be raced and classed, as well as sexed, would have appealed to Gardener, too; for in many ways what she and Stanton hoped to do was align themselves with their elite white male peers and distance themselves from poor women, female immigrants, and women of color” (Hamlin 2007, 153).

The example of Gardener’s nineteenth-century work shows that to succeed with an interdisciplinary and intersectional critique, it is not enough to take issue with the results of empirical methods such as weighing, sizing and mapping alone. Gardener’s story warns us of the dangers of explicitly making only sex/gender difference a subject of discussion, as brain science may also discriminate against the brain of the “other”. In this sense neuroscience today should realize that the concept of innate differences in the brain’s anatomy and brain performance (meaning intelligence) persists, while the (measurement) methods are constantly changing (Staub 2018; Eliot et al., 2021). Today we can observe a rise of statistical and stochastic approaches in brain modelling neuroscience. In order to understand these new methods of empirical measurement and categorization, it is crucial to examine the idea behind these methods and the claim that predictions can be based on the assumptions related to the categories and types employed. Therefore, it is necessary to understand the underlying techniques as well as the empirical statistical process in functional imaging (Fitsch and Friedrich 2018).

## Technical Practices of Difference

In the last few years many scholars have critically investigated the concept of sex/gender research in neuroscience (Bluhm et al., 2012; Kraus 2012; Fine et al., 2013; Schmitz and Höppner 2014; Joel and Fausto-Sterling 2016; Grissom and Reyes 2018; Fausto-Sterling 2019). Nevertheless, in order to address the question of the stage at which sex/gender comes into brain imaging, I will describe the technical conditions in the following. Even though the scanning process itself is not directly linked to sex/gender markers, I want to point to multiple other techniques that inject sex/gender difference into fMRI research. I therefore look at the idea of differentiation that is embedded in the brain imaging method and can be found in the question of the study design, the statistics and the interpretation of the data.

### The Scanning Process

FMRI, as the term suggests, is an imaging method. Imaging procedures are characterized by the fact that they do not translate an original relatum into an image; rather, the technique visualizes a process which simultaneously produces a phenomenon in the first place. The elaborately generated images are the result of an indirect procedure and not, like photography, the depiction of something existing^2^. Brain imaging techniques transform the material brain into a visual medium (Balsamo 1999, 223) by measuring the BOLD signal, which is dependent on the blood oxygenation level and the magnetic susceptibility changes caused by fluctuations in the local oxygen concentration. “It is a direct measurement of the dephasing of spins of water molecules in blood, caused by local differences in magnetic susceptibility. Increased levels of deoxyhemoglobin reduce the BOLD signal; reduced concentrations increase it”. (Roskies 2008, 23).

The technical procedure of fMRI entails the recording of magnetic resonance signals to provide information about specific physical properties of the protons in the brain at a specific location. By changing the physical properties due to biological effects (oxygenation, flow), different local signal intensities are measured under different stimulation conditions and evaluated using statistical methods (t-test, General Linear Model). However, the acquisition of MR images is a non-invasive process that receives signals from the hydrogen protons inside the body through the temporal sequence of magnetic and radio frequency field changes. Subject-specific information is not required, either for the measurement or for the evaluation of the data. At this point in the measurement process the MRI scanner does not evaluate or compare the data, but converts the signals of the hydrogen protons into a digital image. As this happens, certain principles (such as Fourier coding) are exploited. The spatialized voxels are assigned one of 4,095 grey values for the display, which at the same time indicate the activity value of the signal measured there. The scanner is calibrated once on a water phantom, so that no intensity comes out that lies above the scalable range. FMRI produces pure intensity images, meaning that a relative signal is measured rather than an absolute one. It is not important whether the intensity is 900 or 1,100, as long as the other quantities are “in relation”. Since the MR system is a medical diagnostic device, it is possible to enter name, date of birth, sex, weight, height and other information so that a patient can be uniquely identified at a later point in time. Weight is the only information that matters for the tomograph, as it is taken into account to determine the high-frequency radiation deposition in order to prevent harm to the person in the scanner. We should not forget that fMRI has been widely critiqued for the significance it gives to showing “brain activity”. For example, the blood vessels measured for minimal signal changes account for only three percent of a given voxel in the brain (Müller-Jung 2008, N1). In addition, the temporal resolution is very poor: the canonical notion of an optimal BOLD signal assumes neuronal activity that occurs 4–10 s after stimulus exposure (Fitsch, 2012, 282).

### Normalization and Pre-Processing in fMRI

After the scanning process, the data need to be prepared for further analysis and interpretation. Since the measurable signal effect is minor, regions of interest have already been defined in the study design, and the focus will then be placed on these regions in the further evaluation process. Then statistics and standardization come into play: statistical corrections of the data such as noise reduction, correlation analysis, t-test, temporal characteristics of the signal changes (hemodynamic response function); in addition, systemic contaminations that come within a magnetic resonance scanner such as signal drift. Other influencing variables, such as distortions or head movement, are also corrected by using algorithms. To better suppress false positive activations smoothing and clustering methods are used, as well as corrections for multiple comparisons. All of these pre-processing steps refer to statistical standardizations used to prepare the data for analysis. The activation patterns are brought into the form of cartographic representations to identify the areas where a signal change occurred, and these can then be subtracted from each other. Subtractions are used to isolate elements of cognitive processing and generate results by accentuating the differences in the data (Fitsch, 2012).

Normalization in fMRI describes the adjustment of single brains to a stereotactic coordinate system such as Talairach, or MNI, in order to compare the data in the further analysis. The Talairachian reference system is based exclusively on the measurement of the brain of only one woman. For the process of analysis, only one pattern of a region of interest (ROI) is created to avoid single brain fitting, and therefore the brain anatomy has to be aligned to a standard brain to ensure the probability that in each brain the regions of interest are found in the same position (Jäncke 2005). Therefore, not only every single item of anatomical brain data is adjusted to a standardized brain; in addition, the functional data needs to be “normalized” to superimpose the functional data onto the anatomical brain map. In imaging, normalization describes the approximation to stereotactic coordinates and the spatial co-registration of the functional to the anatomical data. Normalization describes the steps in which various brains are matched to a norm brain in order to compare the data obtained from the different subjects. Here “size” becomes a not unimportant parameter in the normalization process: it matters and does not matter at the same time. Size is not an indication of intelligence or thinking activity. But “from the beginning, the search for such sexual dimorphisms in the human brain has been faced with a scaling problem. Recognizing that brain size is related to body size and because human bodies are indeed quite different in size, neuroscientists have had to find ways of comparing brain structures between men and women that won’t merely reflect overall body size” (Eliot et al., 2021, 670). To negate these differences in brain size, which correlate with body and head size and have nothing to do with the individual quality of cognitive performance, “normalizing these measures to individual brain or head size largely eliminates any volume difference between males and females in specific structures” (Eliot et al., 2021, 688).

Initially the default setting has no influence on the further scan procedure, but the individual markers like women/men are nearly always used in fMRI studies even if the option of clicking the checkbox “neutral” is available. This general binary categorization of subjects is highly problematic, as it can be used as a “free category” in your analysis; if you don’t find anything significant in your data, you can still find a publishable finding on gender difference, including false positives, with no further cost for the researchers or any need to collect more data (Bryant et al., 2019). Data analysis in fMRI analysis is based on group comparisons subtracted from each other to find more or less activity in regions of interest. And as the data are already marked in two categories, they can be compared with each other. Every single step to prepare the data for further analysis to make the data comparable is gendered. There is a firmly inscribed male, hetero, white norm here that cannot be easily undermined.

Imaging methods have changed in the last ten years, due to technological developments and especially due to the increasing computing power of computer processors. Following the epistemic alteration from brains as stimulus-response processing systems to brains as prediction machines (Clark 2013), new statistics and the method of computer modelling have become crucial in neuroscience. These computer modelling and machine learning methods are currently being added to established techniques such as functional imaging (Mahfoud et al., 2017). Machine learning is primarily a scoring system that scores the probability of the most likely event (O’Neil 2016), where data “becomes destiny” (Gelman 2018). Modelling has its own epistemological pitfalls, which are different from those of imaging. Yet today less criticism is being directed at the drawbacks of fMRI, such as the stereotactical mapping of behavior and the production of differences through grouping and comparing data, so that data from fMRI studies are being used without being questioned in order to model further with machine learning or Big Data studies.

## Discussion

At the same time as Gardener was writing about brains and education, Anténor (Firmin, 1885) (1850–1911) put forward a fundamental epistemic critique of specific methods for classifying the brain in anthropology. Firmin published his book on the “Equality of Human Races”, *De l’égalité des races humaines: Anthropologie positive*, in 1885. In this work he challenged the racialist anthropometry and craniometry, and racist interpretations of human physical data, of his time. Firmin explicitly criticized the methods of scholars like Paul Broca, who were creating scientific racism using numeric, craniometric tables that showed alleged differences in size and established a white superiority. Reading Gardener together with Firmin’s critique shows in an exemplary way that it is not enough to criticize the gendered results of a so-called empirical method of weighing, sizing and mapping; we also need to look closely at the epistemic ideas behind these methods and to develop multivariate concepts of the brain and its social embeddedness, and of its dependence not only on intra-individual processes but also on intersectional and interpersonal interactions.

Today fMRI data is often analyzed using Big Data and machine learning methods. As Neurofeminism scholars, we can ask how Big Data studies and deep learning can also be helpful in the search for unknown correlates and connections. But as statistics is all about learning from data (Gelman 2018), and statisticians are looking for unexpected patterns using mathematical modelling and data visualizations, one has to be aware of which data are being used to learn from. “The problem of the foundation of statistics is to state a set of principles which entail the validity of all correct statistical inference, and which do not imply that any fallacious inference is valid. But most statistical inference is concerned with a special kind of physical property” (Hacking 1964, 1) Statistical methods become evident in differentiation studies, as the main problem remains: that scientists are still asking the same old question of sex/gender difference (Bluhm 2013; Rippon et al., 2014) and “Why Do We Think Racially?” (Machery and Faucher, 2005; Heinz et al., 2014). Asking about differences, and yet again not only about sex/gender differences but also about “race”, class, and ability differences between brain performances, can be described as a bio-political statement as “it is not driven by new research findings but rather by a priori certainty of the existence of sexed/gendered difference and the heteronormative complementarity inscribed in the very foundations of our society” (Fitsch et al., 2020, 53) This is also true for the categories of “race” and class, and while innovative brain technologies have been prioritized, “the development of innovative brain technologies, perfunctory applications of seemingly objective research tools contribute to structural racism. Thus, neuroscience will benefit from a critical introspection that reassesses existing modalities, techniques, and ontologies retained and relied upon to measure and visualize the brain” (Rollins 2021, 1).

For an interdisciplinary, and perhaps even intersectional, approach to differentiation through brain imaging it is crucial to be aware of the complex technical aspects of neuroimaging research, as they convey the methodological implements for the interpretation of the data. And at the same time, another concept of difference is needed: “The issue here is not only the politics of measure as such, but also the politics of meaning. Our engagements with the neurosciences must therefore begin with the question of how we bring forth difference, and this in itself is the beginning of an ethical response” (Roy 2012, 229) So for a future perspective two issues have to be taken into account. On the one hand, we need to understand the historically implemented concept of “difference” in mathematical calculations and statistical models. And on the other hand, we need to appreciate how these concepts of difference (sex, gender, class, and “race”) are intersectionally intertwined with each other. For interdisciplinary or rather intersectional approaches, we need to ask to what extent the categories of “race” or class have found their way into the statistical measurement strategies of contemporary brain research (Abiodun 2019; Birhane and Guest 2020; Rollins 2021).

## Data Availability

The original contributions presented in the study are included in the article/Supplementary Material, further inquiries can be directed to the corresponding author.

## Footnotes

1 I place “race” in quotation marks, as I truly believe that the category of “race” was invented by scientists to scientifically differentiate humans in a racialized way, which led to racism as it continues to exist today. At the same time the category “race” is necessary to describe the continuities and effects of racism, which are inscribed in the structure of contemporary societies. For further thoughts on the question of “race” in sociology, see the racial formation theory proposed by Michael Omi and Howard Winant (Omi and Winant, 1994).

2 This does not mean that the constructive character of other media such as photography, film, etc. should be denied.
